# Leptospirosis Incidence at Four Sites in Sub-Saharan Africa and South East Asia: An International Multi-Site Hybrid Surveillance Study

**DOI:** 10.1093/ofid/ofag021

**Published:** 2026-03-09

**Authors:** John A Crump, Mathieu Picardeau, Sara A Ajanovic, John Bradley, Justina M Bramugy, Mabvuto Chimenya, Edward W Green, Sham Lal, David C W Mabey, Mayfong Mayxay, Paul N Newton, Ioana D Olaru, Heidi Hopkins, Christian Bottomley, Febrile Illness Evaluation in a Broad Range of Endemicities (FIEBRE) Consortium, Benjamin Amos, Elizabeth A Ashley, Oliver Baerenbold, Stéphanie Baghoumina, Núria Balanza, Tsitsi Bandason, Quique Bassat, Tapan Bhattacharyya, Stuart D Blacksell, Zumilda Boca, Clare I R Chandler, Joseph Chipanga, Anelsio Cossa, Ethel Dauya, Catherine Davis, Xavier de Lamballerie, Justin Dixon, Somyoth Douangphachanh, Audrey Dubot-Pérès, Michelle M Durkin, Nicholas A Feasey, Rashida A Ferrand, Colin Fink, Elizabeth J A Fitchett, Alessandro Gerada, Stephen R Graves, Becca L Handley, Coll D Hutchison, Risara Jaksuwan, Jessica Jervis, Jayne Jones, Kevin C Kain, Suzanne H Keddie, Khamxeng Khounpaseuth, Katharina Kranzer, Khamfong Kunlaya, Pankaj Lal, David G Lalloo, Manophab Luangraj, Yoel Lubell, Eleanor MacPherson, Forget Makoga, Sengchanh Manichan, Florian Maurer, Michael Miles, Polycarp Mogeni, Campos Mucasse, Chelsea Nguyen, Vilayouth Phimolsarnnousith, Chrissy h Roberts, Amphone Sengduangphachanh, Siho Sengsavang, Molly Sibanda, Somvai Singha, John Stenos, Ampai Tanganuchitcharnchai, Hira Tanvir, James E Ussher, Marta Valente, Marie A Voice, Manivanh Vongsouvath, Msopole Wamaka, Shunmay Yeung

**Affiliations:** Centre for International Health, University of Otago, Dunedin, New Zealand; Unité Biologie des Spirochètes, French National Reference Center for Leptospirosis, WHO Collaborating Centre for Reference and Research on Leptospirosis, Institut Pasteur, Paris, France; Centro de Investigação em Saúde de Manhiça, Maputo, Mozambique; ISGlobal—Barcelona Institute for Global Health, Barcelona, Spain; University of Barcelona, Barcelona, Spain; Department of Infectious Disease Epidemiology and International Health, Faculty of Epidemiology and Population Health, London School of Hygiene and Tropical Medicine, London, UK; Centro de Investigação em Saúde de Manhiça, Maputo, Mozambique; Malawi-Liverpool-Wellcome Trust Clinical Research Programme, Blantyre, Malawi; Malawi-Liverpool-Wellcome Trust Clinical Research Programme, Blantyre, Malawi; Department of Clinical Sciences, Liverpool School of Tropical Medicine, Liverpool, UK; Department of Disease Control, Faculty of Infectious and Tropical Diseases, London School of Hygiene and Tropical Medicine, London, UK; Department of Clinical Research, Faculty of Infectious and Tropical Diseases, London School of Hygiene and Tropical Medicine, London, UK; Lao-Oxford-Mahosot Hospital-Wellcome Trust Research Unit (LOMWRU), Microbiology Laboratory, Mahosot Hospital, Vientiane, Lao PDR; Lao-Oxford-Mahosot Hospital-Wellcome Trust Research Unit (LOMWRU), Microbiology Laboratory, Mahosot Hospital, Vientiane, Lao PDR; Nuffield Department of Medicine, Centre for Tropical Medicine and Global Health, University of Oxford, Oxford, UK; Department of Clinical Research, Faculty of Infectious and Tropical Diseases, London School of Hygiene and Tropical Medicine, London, UK; Biomedical Research and Training Institute, Harare, Zimbabwe; Department of Clinical Research, Faculty of Infectious and Tropical Diseases, London School of Hygiene and Tropical Medicine, London, UK; Department of Infectious Disease Epidemiology and International Health, Faculty of Epidemiology and Population Health, London School of Hygiene and Tropical Medicine, London, UK

**Keywords:** Africa, Asia, fever, incidence, leptospirosis

## Abstract

**Background:**

There are few leptospirosis incidence studies despite such estimates being central to accurate burden of disease estimation. We used data from the multicenter Febrile Illness Evaluation in a Broad Range of Endemicities (FIEBRE) study to make leptospirosis incidence estimates from new sites.

**Methods:**

Febrile patients aged ≥2 months in Laos, Malawi, Mozambique, and Zimbabwe were enrolled and underwent standardized clinical and exposure assessment. Acute and convalescent sera were tested by *Leptospira* microscopic agglutination test and acute plasma by *lfb1* polymerase chain reaction (PCR). Participants with ≥4-fold rise in antibody titer between acute and convalescent sample, or *Leptospira* PCR positive for the *lfb1,* had confirmed leptospirosis. Leptospirosis incidence was estimated after adjusting for incomplete enrollment of febrile patients, availability of paired sera, and use of study healthcare facilities by febrile patients based on healthcare utilization data from community controls.

**Results:**

Leptospirosis incidence (95% CI) per 100 000 population per year was 1302 (1011, 1677) in Laos, 1337 (874, 2044) in Malawi, 187 (85, 409) in Mozambique, and could not be calculated for Zimbabwe. Sensitivity analysis restricted to pre-COVID years of 2018 and 2019 produced similar estimates of incidence to that of the whole study period.

**Conclusions:**

Leptospirosis incidence was high at the Laos, Malawi, and Mozambique sites and at the upper end of published incidence estimates from the Asia and Africa regions. We recommend more leptospirosis incidence studies be done in areas lacking data to strengthen leptospirosis global burden of disease estimates and to stimulate progress on diagnosis, management, and control.

## Teaser

Leptospirosis incidence was high at sites in Laos, Malawi, and Mozambique previously lacking data. Leptospirosis is a major cause of febrile illness and more incidence studies are needed to strengthen burden estimates and inform action on diagnosis, management, and prevention.

Leptospirosis was estimated to cause 1.03 million illnesses, 58 900 deaths, and to account for 2.90 disability adjusted life years in 2010 [1, 2]. High quality studies of leptospirosis incidence are needed to improve burden of disease estimates for leptospirosis that in turn inform resource allocation for diagnosis, treatment, and prevention. However, there are few studies of leptospirosis incidence worldwide [1] and data are particularly limited for African countries [3, 4]. Leptospirosis is difficult to distinguish from other causes of febrile illness clinically [5], and reference standard laboratory diagnosis relies on microscopic agglutination testing of paired sera, nucleic acid amplification tests, and bacterial isolation, techniques each with practical diagnostic limitations and that are not widely available in low-resource endemic areas [6].

**Figure 1.**
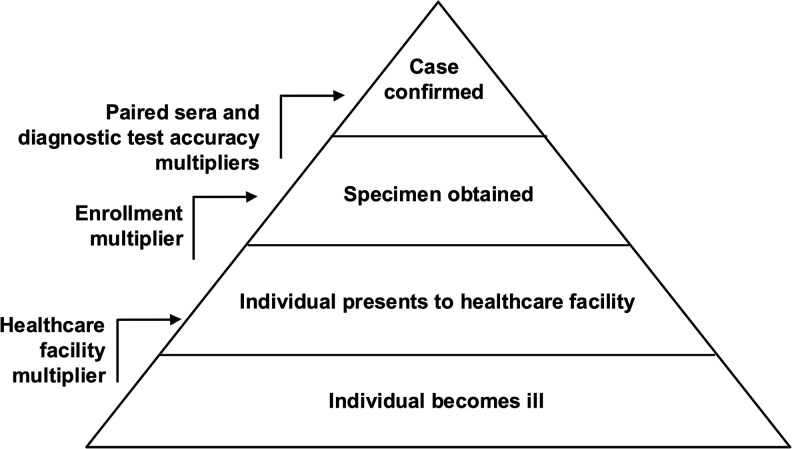
Surveillance pyramid with multipliers to account for incomplete ascertainment at various levels.

Active, population-based surveillance studies have traditionally been used to measure infectious disease incidence, ideally achieved by visiting every household in a defined population regularly to identify those with the clinical syndrome of interest and to collect diagnostic specimens from those affected in the home or to actively refer individuals for testing. Such studies are complex and highly resource intensive. Hybrid surveillance methods, also known as multiplier studies, were developed more than 20 years ago to provide a less resource intensive means to estimate the incidence of acute infectious diseases [7]. Such studies adjust data from sentinel surveillance sites using multipliers to address under-ascertainment, including those derived from healthcare utilization surveys that account for illnesses not presenting to the sentinel sites [7, 8]. The Febrile Illness Evaluation in a Broad Range of Endemicities (FIEBRE) study sought to describe treatable and preventable causes of febrile illness using reference standard diagnostics among inpatients and outpatients at multiple sites with few or no data on causes of fever [9]. Thus, FIEBRE made reference standard leptospirosis diagnostics available and used a standardized diagnostic approach that allow for comparison between study sites. In addition, FIEBRE enrolled community controls that also provided data on healthcare utilization from the catchment populations of sentinel sites to estimate disease incidence using hybrid surveillance methods.

To estimate the incidence of leptospirosis at study sites in the FIEBRE study, we undertook a secondary analysis of the FIEBRE study using data from participants with confirmed lepospirosis and from community healthcare utilization surveys to adjust for under-ascertainment of confirmed leptospirosis at healthcare facilities. Our goal was to provide leptospirosis incidence estimates from locations without such estimates to strengthen evidence to inform resource allocation for disease diagnosis, treatment, and prevention.

## METHODS

The overall design of the FIEBRE study and findings on leptospirosis prevalence and risk factors at FIEBRE sites have been described in detail elsewhere [9, 10]. A brief summary is provided below, including detailed methods for the present analysis.

### Study Sites

The FIEBRE study was conducted at sites four countries selected for having little or no published data on causes of fever and substantial between-site variation in the prevalence of HIV-1/2 and malaria. The sites were Phonghong Vientiane Provincial Hospital, Lao People's Democratic Republic (Lao PDR); Chikwawa District Hospital, Malawi; Manhiça District Hospital, Mozambique; and Sally Mugabe Central Hospital, Parirenyatwa Hospital, Chitungwiza General Hospital, and three primary care clinics in Harare, Zimbabwe.

### Enrollment, Data Collection, and Sample Collection for Febrile Participants

Febrile outpatients and inpatients aged ≥2 months were eligible for enrollment if they had a tympanic or axillary temperature of ≥37.5°C at presentation, and had not been hospitalized or undergone surgery in the previous month. Outpatients were eligible if they resided within the defined catchment area around the health facility at the time of enrollment [9]. For outpatients aged ≥15 years, those with symptoms of lower respiratory infection or of diarrheal disease were excluded. For outpatients aged ≥2 months to <15 years, those with symptoms of diarrheal diseases were excluded. Demographic information, a standardized clinical and exposure history and physical examination was performed on all consented participants and recorded on a case report form.

Whole and EDTA blood were collected on day 0, the day of enrollment, and participants were asked to return on day 28 (acceptable follow-up range: 26–48 days) for collection of convalescent whole blood. Serum and plasma were separated, aliquoted, and stored at −70°C. Samples were shipped on dry ice to the London School of Hygiene and Tropical Medicine, London, United Kingdom, for onward distribution to reference laboratories.

### Laboratory Methods

#### Leptospira Serology

At the Unité Biologie des Spirochètes, French National Reference Center for Leptospirosis, WHO Collaborating Center for Reference and Research on Leptospirosis, Institut Pasteur, Paris, France, acute and convalescent serum was first screened by *Leptospira fainei* serovar Hurstbridge IgM ELISA [11, 12]. Serum from participants that screened positive by ELISA on the acute or convalescent sample proceeded for the *Leptospira* standard microscopic agglutination test (MAT). The MAT panels were based on the World Health Organization recommended list of globally representative reference *Leptospira* strains [13] adjusted to incorporate African and Asian regional isolates (Supplementary Appendix 1) [14].

#### Leptospira Nucleic Acid Amplification Testing and Speciation

Acute plasma from all participants was tested by polymerase chain reaction (PCR) to the *lfb1* pathogenic *Leptospira* target [15]. Among samples positive by *Leptospira lfb1* PCR, PCR products were sequenced for *Leptospira* speciation using established methods [16].

### Case Definitions

We restricted this analysis to participants with rigorously defined confirmed leptospirosis who resided in the study catchment areas [17, 18]. We defined confirmed acute leptospirosis as a participant with paired sera with seroconversion or a ≥ 4-fold rise in MAT antibody titer between acute and convalescent sample, or a participant with paired serum and an acute plasma sample positive by *Leptospira* PCR for the *lfb1* gene target.

### Estimating the Population of Febrile Patients Presenting to Study Facilities

The number of patients presenting with fever to each of the FIEBRE study healthcare facilities was recorded throughout the study period, or estimated from a random sample of calendar days during the study period. This population of febrile patients allowed estimation for each study site of the proportion of eligible patients enrolled into the study.

### Healthcare Utilization Survey

At each site we sought to enroll ≥600 control participants. Control participants were community members in the study site health facilities’ catchment areas, frequency matched one control to two cases to participating outpatients by month of enrollment, age, gender, and geographical location of residence to the outpatients. Potential control participants were approached at their place of residence by study staff with assistance from established community health workers, and enrolled if they or their parent or guardian provided informed consent.

In addition to collection of basic demographic data from control participants, study staff administered questionnaires to capture representative data about healthcare and treatment seeking (Supplementary Appendix 2). Consistent with convention for hybrid surveillance studies [19–21], the control participant, or healthcare decision-maker if the participant was a child, was asked about current and recent fever and care seeking practices for each household member in the event of fever of three or more days duration. The healthcare utilization survey provided an estimate of the proportion of individuals in the community who would present to the study enrollment sites for care in the event of fever of three or more days duration. The fraction of people with fever presenting to a study site was used to estimate the population-based incidence of fever overall, and the incidence of specific causes of fever, in the catchment area of study healthcare facilities.

### Derivation of Multipliers

#### Enrollment Multiplier

The enrollment multiplier was calculated by dividing the number of febrile patients presenting to the study site by the number of participants enrolled $\left(n_{\mathrm{fev}}/n_{\mathrm{enr}}\right)$.

#### Paired Sera Multiplier

The paired sera multiplier was calculated by dividing the number of participants enrolled in the study by the number with paired sera tested by *Leptospira* MAT and acute plasma PCR $\left(n_{\mathrm{enr}}/n_{\mathrm{test}}\right)$.

#### Diagnostic Test Accuracy

In the primary analysis we assumed 100% test sensitivity and specificity as *Leptospira* MAT on paired sera is considered to be the reference standard serologic test for leptospirosis. In a sensitivity analysis, we assumed instead a sensitivity of 93.8% and specificity of 97.3% based on published data on *Leptospira* MAT diagnostic test accuracy [22].

#### Health Facility Multiplier

The health facility multiplier was calculated using responses to healthcare utilization survey questions relating to health-care seeking behavior of the control participant in the event of febrile illness by site healthcare facility. The question was “If you [the control] had a fever that lasted three or more days, where would you usually seek care?’ and “For a fever lasting more than three days, where would you usually go?” We recorded the first, second, third, fourth, and fifth options reported for each queston. Each multiplier was calculated as the reciprocal of the proportion of survey participants who responded that they would attend a particular healthcare facility as their first or second choice healthcare provider $\left(1/{\hat{\;p}}_{\mathrm{seek}}\right)$.

### Population Denominators

Population totals of health facility catchment areas were obtained from the most recent national census or most relevant demographic surveillance period. Population denominators were stratified into age <15 years and age ≥15 years to calculate age-group specific incidence.

### Statistical Analysis

Incidence was estimated by age group, age <15 years, ≥ 15 years, and overall, and by site with the use of the multipliers described above to account for leptospirosis cases that were potentially missed in the stages of reporting (Figure 1).

Specifically, the incidence rate per person year, ${\hat{\lambda }}_{\mathrm{lep}}$, was calculated using the formula:


$${\hat{\lambda }}_{\mathrm{lep}}=\frac{L}{Y}\times \left(\frac{n_{\mathrm{enr}}}{n_{\mathrm{test}}}\right)\times \left(\frac{n_{\mathrm{fev}}}{n_{\mathrm{enr}}}\right)\times \left(\frac{1}{{\hat{\;p}}_{\mathrm{seek}}}\right)$$

where L was the number of confirmed acute leptospirosis cases and *Y* was the number of person-years. The other terms correspond to the multipliers.

This simplifies to:


$${\hat{\lambda }}_{\mathrm{lep}}={\hat{\;p}}_{\mathrm{lep}}\times {\hat{\lambda }}_{\mathrm{fev}}\times \left(\frac{1}{{\hat{\;p}}_{\mathrm{seek}}}\right)$$

where ${\hat{\;p}}_{\mathrm{lep}}=L/n_{\mathrm{test}}$ and ${\hat{\lambda }}_{\mathrm{fev}}=n_{\mathrm{fev}}/\;Y$. Thus, ${\hat{\lambda }}_{\mathrm{lep}}$ can be viewed as the product of three estimates: (1) the proportion positive for leptospirosis among those tested; (2) the incidence of fever cases presenting at a health facility; and (3) the inverse of the proportion of those with fever who seek care. We calculated a 95% confidence interval for $\lambda_{\mathrm{lep}}$ by incorporating uncertainty from each of these three estimates via the delta method (Supplementary Appendix 3) [23].

We conducted two sensitivity analyses. In the first we accounted for the possible surveillance artifact associated with COVID-19 lockdowns and other disruptions from 2020, by restricting to the pre-2020 period. In the second, we adjusted for the sensitivity of 93.8% and specificity of 97.3% of *Leptospira* MAT serology by replacing ${\hat{\;p}}_{\mathrm{lep}}$ in the simplified equation with an adjusted value, ${\hat{\;p}}_{\mathrm{lep}}^{*}$, based on the Rogen-Gladen correction [24]:


$${\hat{\;p}}_{\mathrm{lep}}^{*}=\max\left(\frac{{\hat{\;p}}_{\mathrm{lep}}+\mathrm{sp}-1}{\mathrm{se}+\mathrm{sp}-1},0\right)$$

where se and sp denote the sensitivity and specificity estimates.

### Sample Size

The sample size for this analysis was driven by that of the parent FIEBRE study and is described in detail elsewhere [9].

### Research Ethics

Ethics approval was obtained from the National Ethics Committee for Health Research Committee and the Oxford Tropical Research Ethics Committee for the Laos; the University of Malawi College of Medicine Research and Ethics Committee and the Liverpool School of Tropical Medicine Research Ethics Committee for Malawi; Comité Institucional de Medical Research Bioética para a Saúde do Centro de Investigação em Saúde de Manhiça, and the Comité Nacional de Bioética em Saúde de Moçambique for Mozambique; and the Medical Research Council of Zimbabwe for Zimbabawe. The study was also approved by the research and ethics committee of the London School of Hygiene and Tropical Medicine. Written informed consent is obtained from all participants or their parents or guardians.

## RESULTS

### Leptospirosis Cases

A total of 7851 patients with fever were enrolled in the FIEBRE study, including 1972 (25.1%) in Lao PDR, 1773 (22.6%) in Malawi, 2182 (27.8%) in Mozambique and 1924 (24.5%) in Zimbabwe. The characteristics of the overall FIEBRE cohort and the participants with leptospirosis have been described in detail elsewhere [10]. Of those enrolled, 4503 had paired sera tested by *Leptospira* MAT and acute plasma by *Leptospira lfb1* PCR, including 966 (49.0%) in Lao PDR, 1269 (71.6%) in Malawi, 1191 (54.6%) in Mozambique and 1077 (56.0%) in Zimbabwe. Of those tested, 38 (0.8%) had confirmed leptospirosis based on a positive *Leptospira lfb1* PCR, 96 (2.1%) had confirmed leptospirosis based on paired *Leptospira* MAT serology, and 128 (2.8%) met the confirmed leptospirosis case definition based on either MAT or PCR, including 78 (8.1%) in Lao PDR, 21 (1.7%) in Malawi, 7 (0.6%) in Mozambique, and 22 (2.0%) in Zimbabwe.

### Healthcare Utilization

In the health care utilization survey, 2401 matched control participant households were enrolled, including 485 (20.2%) in Laos, 908 (37.8%) in Malawi, 572 (23.8%) in Mozambique, and 436 (18.2%) in Zimbabwe. Four controls with missing age in Mozambique were excluded from the current analysis. Table 1 show the demographic and co-infection status of FIEBRE controls by site.

**Table 1.** Characteristics of Control Participants, FIEBRE Study, 2018–2021

	Laos, *N* = 485		Malawi, *N* = 908		Mozambique, *N* = 572	
	*n*/*N*	(%)	*n*/*N*	(%)	*n*/*N*	(%)
Age group, years						
0–4	54/485	(11.1)	218/906	(24.1)	186/568	(32.7)
5–14	131/485	(27.0)	179/906	(19.8)	116/568	(20.4)
≥ 15	300/485	(61.9)	509/906	(56.2)	266/568	(46.8)
Sex						
Female	240/485	(49.5)	559/908	(61.6)	367/570	(64.4)
Male	245/485	(50.5)	349/908	(38.4)	203/570	(35.6)
HIV-1/2 status						
Uninfected	0/0	NAN	628/710	(88.5)	486/566	(85.9)
Infected	0/0	NAN	82/710	(11.5)	80/566	(14.1)
Malaria RDT result						
Negative	485/485	(100)	747/858	(87.1)	569/570	(99.8)
Positive	0/485	(0)	111/858	(12.9)	1/570	(0.2)

Abbreviations: NA, not a number; RDT, rapid diagnostic test.

### Multiplier Derivation

#### Enrollment Multiplier

The number of participants enrolled by site and the number of febrile participants presenting by site was 1972 and 16 967 in Laos, 1773 and 77 634 in Malawi, and 2182 and 14 604 in Mozambique, yielding enrollment multipliers of 8.6, 43.8, and 6.7, respectively. Accurate counts of the number of patients presenting with fever to Zimbabwe FIEBRE study healthcare facilities were not available due to COVID-19 disruptions and healthcare worker strikes.

#### Paired Sera Multiplier

The number of participants with paired sera was 966 in Laos, 1269 in Malawi, and 1191 in Mozambique, yielding paired sera multipliers of 2.0, 1.4, 1.8, respectively.

#### Diagnostic Test Accuracy Multiplier

While the primary analysis assumed 100% sensitivity and specificity of *Leptospira* MAT on paired sera, in a sensitivity analysis we assumed a sensitivity of 93.8% and specificity of 97.3% [22].

#### Health Facility Multiplier

The proportion of control participants identifying a FIEBRE study healthcare facility as the first or second choice location that they would seek healthcare in the event of a fever for ≥3 days was 147 (30%) of 485 in Laos, 846 (93%) of 908 in Malawi, and 53 (9%) of 568 in Mozambique, yielding health facility multipliers of 3.3, 1.1, and 10.7, respectively.

### Incidence Calculations

Leptospirosis incidence (95%CI) per 100 000 population per year was 1302 (1,011, 1677) in Laos, 1337 (874, 2044) in Malawi and 187 (85, 409) in Mozambique. We did not calculate incidence for Zimbabwe as accurate counts of the number of patients presenting with fever to Zimbabwe FIEBRE study healthcare facilities were not available. Incidence estimates by age group <15 years, ≥ 15 years and site are shown in Table 2. A sensitivity analysis restricted to pre-COVID years of 2018 and 2019 produced similar estimates of incidence to that of the whole study period (Table 3). A sensitivity analysis adjusted for diagnostic sensitivity and specificity yielded similar estimates in Laos but suggested that apparent incidence in Malawi and Mozambique could be largely attributable to false positives (Table 4).

**Table 2.** Leptospirosis Incidence Calculation by Site and Age Group, FIEBRE Study, 2018–2021

	Confirmed Leptospirosis	Tested	Enrolled	Fever	Seek Care	Study Population	Months	Person Years	Rate	(95% CI)
	(*L*)	($n_{\mathrm{test}}$)	($n_{\mathrm{enr}}$)	($n_{\mathrm{fev}}$)	(${\hat{\;p}}_{\mathrm{seek}}$)			(Y)		
Laos										
≥15 yrs	68	730	1212	11 226	0.35 104/300	115 080	25	239 750.00	1246	(947, 1640)
<15 yrs	10	236	760	5741	0.23 43/185	53 336	25	111 116.67	952	(491, 1845)
All	78	966	1972	16 967	0.3 147/485	168 416	25	350 866.67	1302	(1,011, 1677)
Malawi										
≥15 yrs	17	629	821	37 492	0.93 473/509	23 649	27	53 210.25	2048	(1,280, 3276)
<15 yrs	4	640	952	40 142	0.93 373/399	19 116	29	46 197.00	584	(220, 1552)
All	21	1269	1773	77 634	0.93 846/908	42 765	29	103 348.75	1337	(874, 2044)
Mozambique										
≥15 yrs	5	642	1021	3503	0.12 32/266	128 379	24	256 758.00	89	(35, 226)
<15 yrs	2	549	1161	11 101	0.07 21/302	90 471	28	211 099.00	274	(65, 1160)
All	7	1191	2182	14 604	0.09 53/568	218 850	28	510 650.00	187	(85, 409)

Rate: annual rate per 100 000 population.

$\mathrm{Rate}\;=100,000\;\times \frac{L}{n_{\mathrm{test}}}\times \left(\frac{n_{\mathrm{fev}}}{Y}\right)\times \left(\frac{1}{{\hat{\;p}}_{\mathrm{seek}}}\right).$

**Table 3.** Sensitivity Analysis of Leptospirosis Incidence Calculation by Site and Age Group, FIEBRE Study, Using Pre-COVID Data From 2018–2019

	Confirmed Leptospirosis	Tested	Enrolled	Fever	Seek Care	Study Population	Months	Person Years	Rate	(95% CI)
	(*L*)	($n_{\mathrm{test}}$)	($n_{\mathrm{enr}}$)	($n_{\mathrm{fev}}$)	(${\hat{\;p}}_{\mathrm{seek}}$)			(Y)		
Laos										
≥15 yrs	50	520	890	7842	0.35 104/300	115 080	15	143850	1498	(1103, 2035)
<15 yrs	4	160	538	3141	0.23 43/185	53 336	15	66670	512	(188, 1397)
All	54	680	1428	10983	0.3 147/485	168 416	15	210520	1381	(1033, 1846)
Malawi										
≥15 yrs	16	580	715	24471	0.93 473/509	23 649	18	35473	2046	(1261, 3320)
<15 yrs	4	598	831	21078	0.93 373/399	19 116	18	28674	529	(199, 1406)
All	20	1178	1546	45549	0.93 846/908	42 765	18	64147	1296	(839, 2002)
Mozambique										
≥15 yrs	2	301	441	1576	0.12 32/266	128 379	10	106982	82	(20, 339)
<15 yrs	1	327	705	7642	0.07 21/302	90 471	14	105549	316	(43, 2335)
All	3	628	1146	9218	0.09 53/568	218 850	14	255325	192	(60, 612)

Rate: annual rate per 100 000 population.

$\mathrm{Rate}\;=\;100,000\;\times \frac{L}{n_{\mathrm{test}}}\times \left(\frac{n_{\mathrm{fev}}}{Y}\right)\times \left(\frac{1}{{\hat{\;p}}_{\mathrm{seek}}}\right).$

**Table 4.** Sensitivity Analysis of Leptospirosis Incidence Calculation by Site and Age Group, Adjusted for *Leptospira* MAT Sensitivity of 93.8% and Specificity of 97.3%, FIEBRE Study, 2018–2021

	Confirmed Leptospirosis		Tested	Enrolled	Fever	Seek Care	Population	Months	Person Years	Rate	(95% CI)
	(*L*)	($L_{\mathrm{adj}}$)	($n_{\mathrm{test}}$)	($n_{\mathrm{enr}}$)	($n_{\mathrm{fev}}$)	(${\hat{\;p}}_{\mathrm{seek}}$)			(Y)		
Laos											
≥15 yrs	68	53	730	1212	11 226	0.35 104/300	115 080	25	239 750.00	971	(681, 1384)
<15 yrs	10	17	236	760	5741	0.23 43/185	53 336	25	111 116.67	1618	(298, 8792)
All	78	70	966	1972	16 967	0.3 147/485	168 416	25	350 866.67	1168	(825, 1654)
Malawi											
≥15 yrs	17	0	629	821	37 492	0.93 473/509	23 649	27	53 210.25	0	(0, 361)
<15 yrs	4	0	640	952	40 142	0.93 373/399	19 116	29	46 197.00	0	(0, 438)
All	21	0	1269	1773	77 634	0.93 846/908	42 765	29	103 348.75	0	(0, 191)
Mozambique											
≥15 yrs	5	0	642	1021	3503	0.12 32/266	128 379	24	256 758.00	0	(0, 53)
<15 yrs	2	0	549	1161	11 101	0.07 21/302	90 471	28	211 099.00	0	(0, 411)
All	7	0	1191	2182	14 604	0.09 53/568	218 850	28	510 650.00	0	(0, 80)

Rate: annual rate per 100 000 population.

$\mathrm{Rate}\;=\;100,000\;\times \frac{L_{\mathrm{adj}}}{n_{\mathrm{test}}}\times \left(\frac{n_{\mathrm{fev}}}{Y}\right)\times \left(\frac{1}{{\hat{\;p}}_{\mathrm{seek}}}\right).$

## DISCUSSION

We provide, to the best of our knowledge, the first estimates of leptospirosis incidence from sites in Lao PDR, Malawi, and Mozambique, adding considerably to the limited number of available estimates of leptospirosis incidence from low-resource settings. We show that leptospirosis incidence exceeded 100 per 100 000 population per year at sites in three countries, highlighting considerable illness in these previously unstudied locations.

We estimated that incidence of leptospirosis was significantly higher in the Lao PDR and Malawi sites than the Mozambique site during the study period. While leptospirosis incidence in Laos and Malawi may be stably higher than in Mozambique, it is also possible that differences represent fluctuations in incidence over time, as has been observed elsewhere [19]. We have shown that work in rice fields was substantially more common at the Lao PDR site than in the African sites [10]. Additionally, the Malawi study site of Chikwawa is located in a rural, low-lying area adjacent to and including the flood plain of the Shire River, a major tributary to the Zambezi River. It could therefore be speculated that both the Laos and Malawi study catchment areas provide substantial risk for exposure to moist soil potentially contaminated with *Leptospira* [10]. Notably, the FIEBRE leptospirosis incidence estimates were at the high end of the range of those identified by a systematic review of the World Health Organization Leptospirosis Burden Epidemiology Reference Group (LERG) [1]. Among studies identified in the LERG review from Africa incidence per 100 000 per year was 69 in Cameroon 1975–1976 [25], 101 in the Seychelles in 1995–1996 [26], and 160 in Wonji, Ethiopia 2003 [27]. Among studies from countries neighboring Lao PDR, incidence per 100 000 per year was 5 from a study from Takeo Province, Cambodia 2003 [28], 15 from Mahasarakam Province, Thailand 1996 [29], 7 from Nakornratchasrima, Thailand 1998 [30], 2 from Kamphaeng Phet, Thailand 1998–2003 [31], and 10 from Thailand national data 1994–2003 [32]. However, the LERG review included studies that measured crude incidence, often from passive surveillance, and as such likely represent underestimates of actual leptospirosis incidence [7, 8]. More data from active population-based or hybrid surveillance studies would be useful to understand the true incidence of leptospirosis.

We did not observe significant differences in leptospirosis incidence among those aged ≥15 years compared with those aged <15 years at any study site. There were statistically non-significant trends toward higher incidence in the older age groups in Laos and Malawi, and younger age groups in Mozambique. Review of global leptospirosis data indicates that risk for disease tends to be higher in adults than in children [1], likely associated with occupational exposures involving direct contact with infected animals and indirect contract with contaminated environments [6].

Our study had a number of limitations. While hybrid surveillance is a widely accepted and used method for estimating the incidence of acute infectious diseases [7, 8], factors such as differential healthcare seeking among risk groups mean that it is unlikely to yield incidence estimates that are as accurate as active, population-based surveillance. The FIEBRE study took place during the COVID-19 pandemic, resulting in disruptions to health systems that may have influenced our sentinel surveillance for leptospirosis and the healthcare utilization survey among controls. Indeed, in Zimbabwe the COVID-19 disruptions and healthcare worker strikes meant that it was not possible to estimate leptospirosis incidence there. However, for the remaining sites we explored the impact of the COVID-19 pandemic through a sensitivity analysis comparing incidence estimates from the pre-pandemic with those of the post-pandemic period and found no significant differences. Our diagnostic strategy was also a source of limitations. If the *L. fainei* serovar Hurstbridge IgM ELISA screening test used prior to *Leptospira* MAT had sensitivity <100%, we may have failed to detect some participants with leptospirosis resulting in under-estimation of disease incidence. Furthermore, incidence estimates are also sensitive to assumptions about the diagnostic accuracy of MAT, particularly its specificity. Because the prevalence of true cases is low, even a small number of false positives can substantially influence the estimates. Leptospirosis is a zoonosis with a complex ecology and incidence varies in both time and place. Therefore, our incidence estimates may not be generalizable to other locations in Asia or Africa, nor even to the same locations in different time periods.

We show that leptospirosis incidence was high in Lao PDR, Malawi, and Mozambique, countries that hitherto lack leptospirosis incidence estimates. The very high incidence observed in Laos and Malawi may relate to rice field exposure and exposure to moist flood plain environments, respectively. Our incidence estimates are at the high end of reported data and exceed those identified by the LERG systematic review of incidence studies, perhaps in part due to our use of the hybrid surveillance design to approximate incidence observed in active, population-based incidence studies. We recommend that leptospirosis be included and regularly updated in global burden of disease estimates to draw the attention of policy makers and those willing to invest in its diagnosis, management, and control.

## Supplementary Material

ofag021_Supplementary_Data

## Supplementary Data

Supplementary materials are available at *Open Forum Infectious Diseases* online. Consisting of data provided by the authors to benefit the reader, the posted materials are not copyedited and are the sole responsibility of the authors, so questions or comments should be addressed to the corresponding author.
