# Supplementary material for: Leptospirosis Incidence at Four Sites in Sub-Saharan Africa and South East Asia: An International Multi-Site Hybrid Surveillance Study
Source: Open Forum Infect Dis. 2026 Mar 9;13(3):ofag021. doi: 10.1093/ofid/ofag021 (PMC12970525; doi:10.1093/ofid/ofag021)
Supplement: ofag021_Supplementary_Data [file ofag021_supplementary_data.zip › Lepto_FIEBRE_Incidence_SupplAppendix1_20Mar2025.docx]

**Supplementary Appendix 1. Febrile Illness Evaluation in a Broad Range of Endemicities (FIEBRE) *Leptospira* standard microscopic agglutination test MAT panels**

The Africa MAT panel was used to test samples from Malawi, Mozambique, and Zimbabawe, and included 18 *Leptospira* strains belonging to 11 serogroups (representative species and serovar name; numeric or alpha-numeric serovar alias represent internal strain numbers of the French National Reference Center for Leptospirosis): Australis (represented by *L. interrogans* serovar Australis, *L. interrogans* serovar Lora); Autumnalis (represented by *L. kirschneri* serovar 202001911); Ballum (*L. borgpetersenii* serovar Arborea, *L. borgpetersenii* serovar Kenya); Canicola (*L. interrogans* serovar Canicola, *L. interrogans* serovar Kuwait); Grippotyphosa (*L. kirschneri* serovar 201600670); Hebdomadis (*L. interrogans* serovar Hebdomadis); Icterohaemorrhagiae (*L. interrogans* serovar Copenhageni, *L. kirschneri* serovar Sokoine); Mini (*L. borgpetersenii* serovar 200801925, *L. borgpetersenii* serovar 201501056); Pyrogenes (*L. borgpetersenii* serovar 202001912, *L. interrogans* serovar 201700941); Pomona (*L. borgpetersenii* serovar 201501070, *L. interrogans* serovar Pomona); and Sejroë (*L. borgpetersenii* serovar Sejroë).

The Asia MAT panel was used to test samples from Laos, and included 16 *Leptospira* strains belonging to 13 serogroups (representative species and serovar name; numeric serovar designations represent internal strain numbers of the French National Reference Center for Leptospirosis): Australis (represented by *L. interrogans* serovar 201801194); Autumnalis (*L. interrogans* serovar Autumnalis, *L. interrogans* serovar 201801758); Bataviae (*L. interrogans* serovar 201801193); Canicola (*L. interrogans* serovar 201801169); Celledoni (*L. weilii* serovar 201801177); Grippotyphosa (*L. interrogans* serovar 201801158, *L. interrogans* serovar 201801175); Javanica (*L. borgpetersenii* serovar 201801754); Hebdomadis (*L. weilii* serovar 201801206); Icterohaemorrhagiae (*L. interrogans* serovar 201801765, *L. interrogans* serovar Icterohaemorrhagiae); Mini (*L. weilii* serovar 201801198); Pomona (*L. interrogans* serovar 201801207); Pyrogenes (*L. interrogans* serovar Pyrogenes); and Sejroë (*L. borgpetersenii* serovar Sejroë).
