# Supplementary material for: Leptospirosis Incidence at Four Sites in Sub-Saharan Africa and South East Asia: An International Multi-Site Hybrid Surveillance Study
Source: Open Forum Infect Dis. 2026 Mar 9;13(3):ofag021. doi: 10.1093/ofid/ofag021 (PMC12970525; doi:10.1093/ofid/ofag021)
Supplement: ofag021_Supplementary_Data [file ofag021_supplementary_data.zip › Lepto_FIEBRE_Incidence_SupplAppendix2_20Mar2025.docx]

**Supplementary Appendix 2. Febrile Illness Evaluation in a Broad Range of Endemicities (FIEBRE) study control participant case report form questions on healthcare utilization**

**ODK Form ID: mwcon9 , ODK Form version: 2018071210 , ODK Excel filename: FIEBREcontrolq_v10MW_13_07_2018_JD_07_08_2018_SL_v9.xlsx**

**Control CRF**

| **Question** | **Question hint** | **Response options** |
| --- | --- | --- |
| Today's date |  | Date/Time |
| eCRF Start time |  | Date/Time |
| Tablet ID |  |  |
| subscriberid |  |  |
| simserial |  |  |
| phonenumber |  |  |
| Welcome to the Electronic FIEBRE CONTROL data collection form.    Please make sure that all the appropriate materials and supplies are ready. <br> <br> This includes hard copies of the control information sheet and consent forms, participant ID stickers, blood collection materials, pharyngeal swabs, and tubes. |  |  |
| An ODK calculated value |  | Calculated value |
| An ODK calculated value |  | Calculated value |
| Site information |  |  |
| Please select the country |  | Select one: Laos, Malawi, Mozambique, Myanmar, Zimbabwe |
| Is this the current date?  <span style="color:red"> ${c_date_today} </span> |  | Select one: Yes, No |
| Is this the current time?  <span style="color:red">${c_time_today}</span> |  | Select one: Yes, No |
| Please enter your health worker name |  | Select one: Barbara Njamwaha, Brigitte Denis, Ed Green, Fletcher Nangupeta, Frank Mbalume, Neema Nyakuleha, James Chinseu, Kestings Gwedemula, Mabvuto Chimenya, Maggie Thole, Kenneth Chizani, Patrick Hussein, Wamaka Msopole. |
| Enter the correct date |  | Date/Time |
| Enter the correct time |  |  |
| Take the GPS co-ordinates of the enrolled participant's location | Ensure this is taken outdoors | GPS |
| _Matched control participant_  In the next screen, complete the information for the matched control and enter the matched control's participant ID |  |  |
| (To the interviewer: Have you and/or the control participant (or the parent/guardian) read the appropriate information sheets?) |  | Select one: Yes, No |
| Please go back and read the information sheets. |  |  |
| ***(To the interviewer: Has the participant provided consent and agrees to participate in the study?)*** | Ensure all the appropriate boxes on the consent form have been ticked and the consent form has been signed or marked with a finger print if appropriate | Select one: Yes, No |
| ***(To the interviewer: Have you and/or the control participant (or the parent/guardian) read the sample collection and storage information sheet)*** |  | Select one: Yes, No |
| Please go back and read the sample collection and storage information sheets. |  |  |
| ***(To the interviewer: Does the control participant (or the parent/guardian) agree to have their samples stored for future use?)*** | Ensure all the appropriate boxes on the consent form have been ticked and the future use consent form has been signed or marked with a finger print if appropriate | Select one: Yes, No |
| Once the consent form is completed, place a participant ID sticker on the consent form. |  |  |
| Once the future use and storage consent form is completed, place a participant ID sticker on the future use consent form. |  |  |
| Control consent |  |  |
| 2.0a: Use the tablet's to scan the QR code in the top right hand corner of the signed consent form |  | barcode |
| *(To the interviewer: Did the QR Code scan properly?)* | If no then manually type the ID on the next screen | Select one: Yes, No |
| 2.0b: Type the participant ID number in the format AB12345 | The ID can be read from the strip of ID stickers. | string value |
| Please go back and type the participant ID or scan the QR code on the consent form |  |  |
| An ODK calculated value |  | Calculated value |
| Control consent future use and storage |  |  |
| 3.0a: Use the tablet's camera to scan the QR code in the top right hand corner of the signed future use and storage consent form. |  | barcode |
| *(To the interviewer: Did the QR Code scan properly?)* | If no then manually type the ID | Select one: Yes, No |
| 3.0b: Type the participant ID number | The ID can be read from the strip of ID stickers | string value |
| Please go back and type the participant ID or scan the QR code on the consent form |  |  |
| An ODK calculated value |  | Calculated value |
| An ODK calculated value |  | Calculated value |
| The participant IDs scanned or typed on the participant consent form and the participant sample future use form do not match. Please go back to check the participant IDs on each consent form, and enter the correct participant ID |  |  |
| Control eCRF for ${patient_id_calculate} |  |  |
| Section 1: Demographics |  |  |
| Location of control assessment |  | Select one: Control’s home, Study health facility, Other |
| If the location is the control's home, take the GPS co-ordinates | Ensure this is taken outdoors | GPS |
| If other, state where the asessment is taking place |  | Text |
| What is the participant's date of birth (DOB)? | If the respondent does not know the date of birth leave blank and enter their age on the next screen. |  |
| Day | Enter a number between 1 and 31 | Integer value |
| Month |  | Select one: Jan, Feb, Mar, Apr, May, Jun, Jul, Aug, Sep, Oct, Nov, Dec |
| Year |  | Integer value |
| Is the control participant less than 12 months old? |  | Select one: Yes, No |
| How old is the control participant in weeks? | Enter the number of weeks, which should be <=52 weeks. | Integer value |
| Is the control participant under 5 years old? |  | Select one: Yes, No |
| How old is the control participant in months? | Enter the number of months, which should be <60 months. | Integer value |
| How old is the control participant in years? | Enter the age in years between 5 and 120 years. | Integer value |
| What is the control participant's gender? |  | Select one: Male, Female |
| What is the control participant’s self-reported ethnicity? |  | Select one: Shona, Ndebele, Other ethnicity, Patient refused to disclose, Chewa, Mang'anja, Lomwe, Yao, Ngoni, Tumbuka, Nyanja, Sena, Tonga, Ngonde, Other ethnicity, Patient refused to disclose, Katang, Vietnamese, Bru, Kuy, Laven, Oi, Katu, Lave, Ngae, Khmer, Jeh, Khlor, Ir, Alak, Bo, Halang Doan, Hung or Tum, Khua, Aheu, Maleng, Arem, Chut, Kri, Lavae/Brao, Lavi, Mon, Nguon, Nyaheun, Ong, Pakoh, Phong, Sadang, Salang, Sapuan, Makong, Sou, Souei, Taliang, Ta-oi, Yae, Patient refused to disclose, Other ethnicity, Makua, Sena, Shona, Shangaan, Makonde, Yao, Swahili, Tonga, Chopi, Nguni, Other ethnicity, Patient refused to disclose, Shangaan, Kayin, Kayar, Mon, Rakhine, Shan, Other ethnicity, Patient refused to disclose |
| Is the respondent the same person as the control participant? | The respondent is the person answering the interview questions. | Select one: Yes, No |
| What is the respondent's relationship to the control? |  | Select one: Mother, Father, Aunt, Uncle, Grandmother, Grandfather, Cousin, Sister, Brother, Guardian, Spouse, Other |
| What is the respondent's date of birth (DOB)? | If the respondent does not know the date of birth leave blank and enter their age on the next screen. |  |
| Day | Enter a number between 1 and 31 | Integer value |
| Month |  | Select one: Jan, Feb, Mar, Apr, May, Jun, Jul, Aug, Sep, Oct, Nov, Dec |
| Year |  | Integer value |
| How old is the respondent in years? | Enter the age in years. | Integer value |
| What is the respondent's gender? |  | Select one: Male, Female |
| Do you [the control] have a fever today? |  | Select one: Yes, No |
| Enter the control's ear temperature | In degrees Celsius (°C) to 1 decimal place. If you cannot obtain the temperature, enter 999.  If the control reports fever and/or has an elevated temperature, encourage her/him to seek care if desired | decimal value |
| Healthcare utilisation |  |  |
| Section 5: Healthcare utilisation |  |  |
| If you [the control] had a fever that lasted _less than 3 days_, where would you usually seek care? |  |  |
| Traditional healers |  | Select one: I would go |
| Drug shops |  | Select one: I would go |
| Public health facility |  | Select one: I would go |
| Private health facility |  | Select one: I would go |
| Pharmacy |  | Select one: I would go |
| Church |  | Select one: I would go |
| Chikwawa District Hospital |  | Select one: I would go |
| Harare Central Hospital |  | Select one: I would go |
| Budiriro Poly Clinic |  | Select one: I would go |
| Chitungwiza General Hospital |  | Select one: I would go |
| Other |  | Select one: I would go |
| State other place: |  | Text |
| For a fever lasting _less than 3 days_, where would you usually go first, second, third, fourth, fifth? |  |  |
| Traditional healers |  | Select one: First, Second, Third, Fourth, Fifth |
| Drug shops |  | Select one: First, Second, Third, Fourth, Fifth |
| Public health facility |  | Select one: First, Second, Third, Fourth, Fifth |
| Private health facility |  | Select one: First, Second, Third, Fourth, Fifth |
| Pharmacy |  | Select one: First, Second, Third, Fourth, Fifth |
| Church |  | Select one: First, Second, Third, Fourth, Fifth |
| Chikwawa District Hospital |  | Select one: First, Second, Third, Fourth, Fifth |
| Harare Central Hospital |  | Select one: First, Second, Third, Fourth, Fifth |
| Budiriro Poly Clinic |  | Select one: First, Second, Third, Fourth, Fifth |
| Chitungwiza General Hospital |  | Select one: First, Second, Third, Fourth, Fifth |
| Other place: ${seek_care_other_less} |  | Select one: First, Second, Third, Fourth, Fifth |
| If you [the control] had a fever that lasted _3 days or longer_, where would you usually seek care? |  |  |
| Traditional healers |  | Select one: I would go |
| Drug shops |  | Select one: I would go |
| Public health facility |  | Select one: I would go |
| Private health facility |  | Select one: I would go |
| Pharmacy |  | Select one: I would go |
| Church |  | Select one: I would go |
| Chikwawa District Hospital |  | Select one: I would go |
| Harare Central Hospital |  | Select one: I would go |
| Budiriro Poly Clinic |  | Select one: I would go |
| Chitungwiza General Hospital |  | Select one: I would go |
| Other |  | Select one: I would go |
| State other place: |  | Text |
| For a fever lasting _3 days or longer_, where would you usually go; first, second, third, fourth, fifth? |  |  |
| Traditional healers |  | Select one: First, Second, Third, Fourth, Fifth |
| Drug shops |  | Select one: First, Second, Third, Fourth, Fifth |
| Public health facility |  | Select one: First, Second, Third, Fourth, Fifth |
| Private health facility |  | Select one: First, Second, Third, Fourth, Fifth |
| Pharmacy |  | Select one: First, Second, Third, Fourth, Fifth |
| Church |  | Select one: First, Second, Third, Fourth, Fifth |
| Chikwawa District Hospital |  | Select one: First, Second, Third, Fourth, Fifth |
| Harare Central Hospital |  | Select one: First, Second, Third, Fourth, Fifth |
| Budiriro Poly Clinic |  | Select one: First, Second, Third, Fourth, Fifth |
| Chitungwiza General Hospital |  | Select one: First, Second, Third, Fourth, Fifth |
| Other place: ${seek_care_other_more} |  | Select one: First, Second, Third, Fourth, Fifth |
| How many other people are in the control’s household? | Define “household” as all the people who eat the evening meal together most nights of the week. If the control participant usually eats alone, enter “0” | Integer value |
| Household member 1 (etc, for other members): |  |  |
| For the 1st household member: |  |  |
| What is their date of birth (DOB)? | If the respondent does not know the date of birth leave blank and enter their age on the next screen. |  |
| Day | Enter a number between 1 and 31 | Integer value |
| Month |  | Select one: Jan, Feb, Mar, Apr, May, Jun, Jul, Aug, Sep, Oct, Nov, Dec |
| Year |  | Integer value |
| Is the household member less than 12 months old? |  | Select one: Yes, No |
| How old is the household member in weeks? | Enter the number of weeks, which should be <=52. | Integer value |
| Is the household member under 5 years old? |  | Select one: Yes, No |
| How old is the household member in months? | Enter the number of months, which should be <60. | Integer value |
| How old is the household member in years? | Enter the age in years. | Integer value |
| What is the household member's gender? |  | Select one: Male, Female |
| If household member 1 had a fever that lasted _less than 3 days_, where would they usually seek care?  (if the household member doesn't know, leave each of these responses blank) |  |  |
| Traditional healers |  | Select one: I would go |
| Drug shops |  | Select one: I would go |
| Public health facility |  | Select one: I would go |
| Private health facility |  | Select one: I would go |
| Pharmacy |  | Select one: I would go |
| Church |  | Select one: I would go |
| Chikwawa District Hospital |  | Select one: I would go |
| Harare Central Hospital |  | Select one: I would go |
| Budiriro Poly Clinic |  | Select one: I would go |
| Chitungwiza General Hospital |  | Select one: I would go |
| Other |  | Select one: I would go |
| State other place: |  | Text |
| For a fever lasting _less than 3 days_, where would you usually go first, second, third, fourth, fifth? |  |  |
| Traditional healers |  | Select one: First, Second, Third, Fourth, Fifth |
| Drug shops |  | Select one: First, Second, Third, Fourth, Fifth |
| Public health facility |  | Select one: First, Second, Third, Fourth, Fifth |
| Private health facility |  | Select one: First, Second, Third, Fourth, Fifth |
| Pharmacy |  | Select one: First, Second, Third, Fourth, Fifth |
| Church |  | Select one: First, Second, Third, Fourth, Fifth |
| Chikwawa District Hospital |  | Select one: First, Second, Third, Fourth, Fifth |
| Harare Central Hospital |  | Select one: First, Second, Third, Fourth, Fifth |
| Budiriro Poly Clinic |  | Select one: First, Second, Third, Fourth, Fifth |
| Chitungwiza General Hospital |  | Select one: First, Second, Third, Fourth, Fifth |
| Other place: ${h1_seek_care_other_less} |  | Select one: First, Second, Third, Fourth, Fifth |
| If household member 1 had a fever that lasted _3 days or longer_, where would you usually seek care?  (if the household member doesn't know, leave each of these responses blank) |  |  |
| Traditional healers |  | Select one: I would go |
| Drug shops |  | Select one: I would go |
| Public health facility |  | Select one: I would go |
| Private health facility |  | Select one: I would go |
| Pharmacy |  | Select one: I would go |
| Church |  | Select one: I would go |
| Chikwawa District Hospital |  | Select one: I would go |
| Harare Central Hospital |  | Select one: I would go |
| Budiriro Poly Clinic |  | Select one: I would go |
| Chitungwiza General Hospital |  | Select one: I would go |
| Other |  | Select one: I would go |
| State other place: |  | Text |
| For a fever lasting _3 days or longer_, where would you usually go; first, second, third, fourth, fifth? |  |  |
| Traditional healers |  | Select one: First, Second, Third, Fourth, Fifth |
| Drug shops |  | Select one: First, Second, Third, Fourth, Fifth |
| Public health facility |  | Select one: First, Second, Third, Fourth, Fifth |
| Private health facility |  | Select one: First, Second, Third, Fourth, Fifth |
| Pharmacy |  | Select one: First, Second, Third, Fourth, Fifth |
| Church |  | Select one: First, Second, Third, Fourth, Fifth |
| Chikwawa District Hospital |  | Select one: First, Second, Third, Fourth, Fifth |
| Harare Central Hospital |  | Select one: First, Second, Third, Fourth, Fifth |
| Budiriro Poly Clinic |  | Select one: First, Second, Third, Fourth, Fifth |
| Chitungwiza General Hospital |  | Select one: First, Second, Third, Fourth, Fifth |
| Other place: ${h1_seek_care_other_more} |  | Select one: First, Second, Third, Fourth, Fifth |
| You have reached the end of the eCRF. Please ensure you have completed the following actions:    1) Collect the blood and pharyngeal swab samples from the control participant  2) Enter the HIV test result if done  3) Mark the form as finalised <br> <br> Thank you |  |  |
| The patient has not consented to the study. Continue to end the CRF and please ensure the following actions are done  1) Ensure the eCRF is marked as finalised  2) Save and exit the CRF |  |  |
