# Supplementary material for: Leptospirosis Incidence at Four Sites in Sub-Saharan Africa and South East Asia: An International Multi-Site Hybrid Surveillance Study
Source: Open Forum Infect Dis. 2026 Mar 9;13(3):ofag021. doi: 10.1093/ofid/ofag021 (PMC12970525; doi:10.1093/ofid/ofag021)
Supplement: ofag021_Supplementary_Data [file ofag021_supplementary_data.zip › Lepto_FIEBRE_Incidence_SupplAppendix3_23Oct2025.docx]

**Supplementary Appendix 3. Febrile Illness Evaluation in a Broad Range of Endemicities (FIEBRE) calculation of 95% confidence intervals and adjustment for test accuracy for leptospirosis incidence estimates**

**95% confidence interval calculation**

A confidence interval for the leptospirosis incidence rate can be obtained by expressing the rate as a product of three estimates and determining the uncertainty in these estimates.

The incidence rate, $\hat{\lambda}_{lep}$, is estimated using the formula (1):

$\hat{\lambda}_{lep}=\frac{L}{Y}\times\left( \frac{n_{enr}}{n_{test}} \right)\times\left( \frac{n_{fev}}{n_{enr}} \right)\times\left( \frac{1}{\hat{p}_{seek}} \right)$

(1)

where:

$L$ = no. of leptospirosis-positive FIEBRE participants with fever

$Y$ = no. of person years (catchment population size $\times$ study duration)

$n_{test}$ = no. of enrolled participants with fever tested for leptospirosis

$n_{enr}$ = no. of enrolled participants with fever in the FIEBRE study

$n_{fev}$ = no. of persons with fever presenting at a health facility

$\hat{p}_{seek}$ = estimated proportion of fever cases who seek care

Alternatively, $\hat{\lambda}_{lep}$ can be written as

(2)

$$\hat{\lambda}_{lep}=\hat{p}_{lep}\times\hat{\lambda}_{fev}\times\left( \frac{1}{\hat{p}_{seek}} \right)$$

where $\hat{p}_{lep}={L/n}_{test}$ and $\hat{\lambda}_{fev}=$ $n_{fev}/ Y$. In other words, $\hat{\lambda}_{lep}$ is the product of three estimates: (i) the proportion positive for leptospirosis among those tested; (ii) the incidence of fever presenting at a health facility; and (iii) the proportion of those with fever who seek care.

Taking logs of equation (2) above,

$$\log\left( \hat{\lambda}_{lep} \right)=\log\left( \hat{p}_{lep} \right)+\log\left( \hat{\lambda}_{fev} \right)-log(\hat{p}_{seek})$$

The variance is given by:

$$Var[log \left( \hat{\lambda}_{lep} \right)]=Var[log \left( \hat{p}_{lep} \right)]+Var[log \hat{\lambda}_{fev}]+Var[log \left( \hat{p}_{seek} \right)]$$

Using the delta method, $\mathrm{Var}\left[ \log\left( X \right) \right]\approx\mathrm{Var}[X]/E\left[ X \right]^{2}$ therefore

$$Var[log \left( \hat{\lambda} \right)]\approx\frac{Var[\hat{p}_{lep} ]}{E\left[ \hat{p}_{lep} \right]^{2}}+\frac{Var[\hat{\lambda}_{fev} ]}{E\left[ \hat{\lambda}_{fev} \right]^{2}}+\frac{Var[\hat{p}_{seek} ]}{E\left[ \hat{p}_{seek} \right]^{2}}$$

Plugging in estimates for each quantity we obtain:

$Var[log \left( \hat{\lambda}_{lep} \right)]\approx\frac{\left( 1-\hat{p}_{lep} \right)}{n_{test}\hat{p}_{lep}}+\frac{1}{n_{fev}}+$ $\frac{\left( 1-\hat{p}_{seek} \right)}{n\hat{p}_{seek}}$

where $n$ is the number controls interviewed in the health facility utilization survey.

Finally, the standard error is:

$std err=\sqrt{\frac{\left( 1-\hat{p}_{lep} \right)}{n_{test}\hat{p}_{lep}}+\frac{1}{n_{fev}}+ \frac{\left( 1-\hat{p}_{seek} \right)}{n\hat{p}_{seek}}}$

and the 95% confidence interval for $\hat{\lambda}$ is given by exp[log($\hat{\lambda})\pm1.96\times std err]$.

**Adjusting for accuracy of the test**

To adjust for the sensitivity and specificity of the diagnostic test, we replace $\hat{p}_{lep}$ in equation (2) with an adjusted value $\hat{p}_{lep}^{*}$:

$$\hat{p}_{lep}^{*}=max\left( \frac{\hat{p}_{lep}+sp-1}{se+sp-1},0 \right)$$

where $se$ and $sp$ are the sensitivity and specificity of the test. This adjustment follows the Rogan–Gladen correction, which accounts for imperfect test accuracy when estimating disease prevalence.

Assuming $se$ and $sp$ are fixed, the standard error for the adjusted estimate of log incidence is

$$std err=\sqrt{\frac{\hat{p}_{lep}\left( 1-\hat{p}_{lep} \right)}{{n_{test}\left( \hat{p}_{lep}+sp-1 \right)}^{2}}+\frac{1}{n_{fev}}+ \frac{\left( 1-\hat{p}_{seek} \right)}{n\hat{p}_{seek}}}$$
